# Editorial: Nucleic Acid Polymerases: The Two-Metal-Ion Mechanism and Beyond

**DOI:** 10.3389/fmolb.2022.948326

**Published:** 2022-07-13

**Authors:** Janice D. Pata, Y. Whitney Yin, Indrajit Lahiri

**Affiliations:** ^1^ Wadsworth Center, New York State Department of Health, Albany, NY, United States; ^2^ Department of Biomedical Sciences, School of Public Health, University at Albany, Albany, NY, United States; ^3^ Department of Biochemistry and Molecular Biology, University of Texas Medical Branch, Galveston, TX, United States; ^4^ Department of Biological Sciences, Indian Institute of Science Education and Research Mohali, Punjab, India

**Keywords:** DNA polymerase, RNA polymerase, reverse transcriptase, cellular polymerase, viral polymerase

Nucleic acid polymerases are essential for all forms of life, performing diverse functions from genome replication and repair to the transcription of a wide variety of RNAs. Although these enzymes differ widely in substrate specificity, efficiency, accuracy, and evolutionary origin, they all catalyze the same nucleotidyltransferase reaction. This eBook on “*Nucleic Acid Polymerases: The Two-Metal-Ion Mechanism and Beyond*” highlights both the similarities and differences among these enzymes.

**FIGURE 1:**
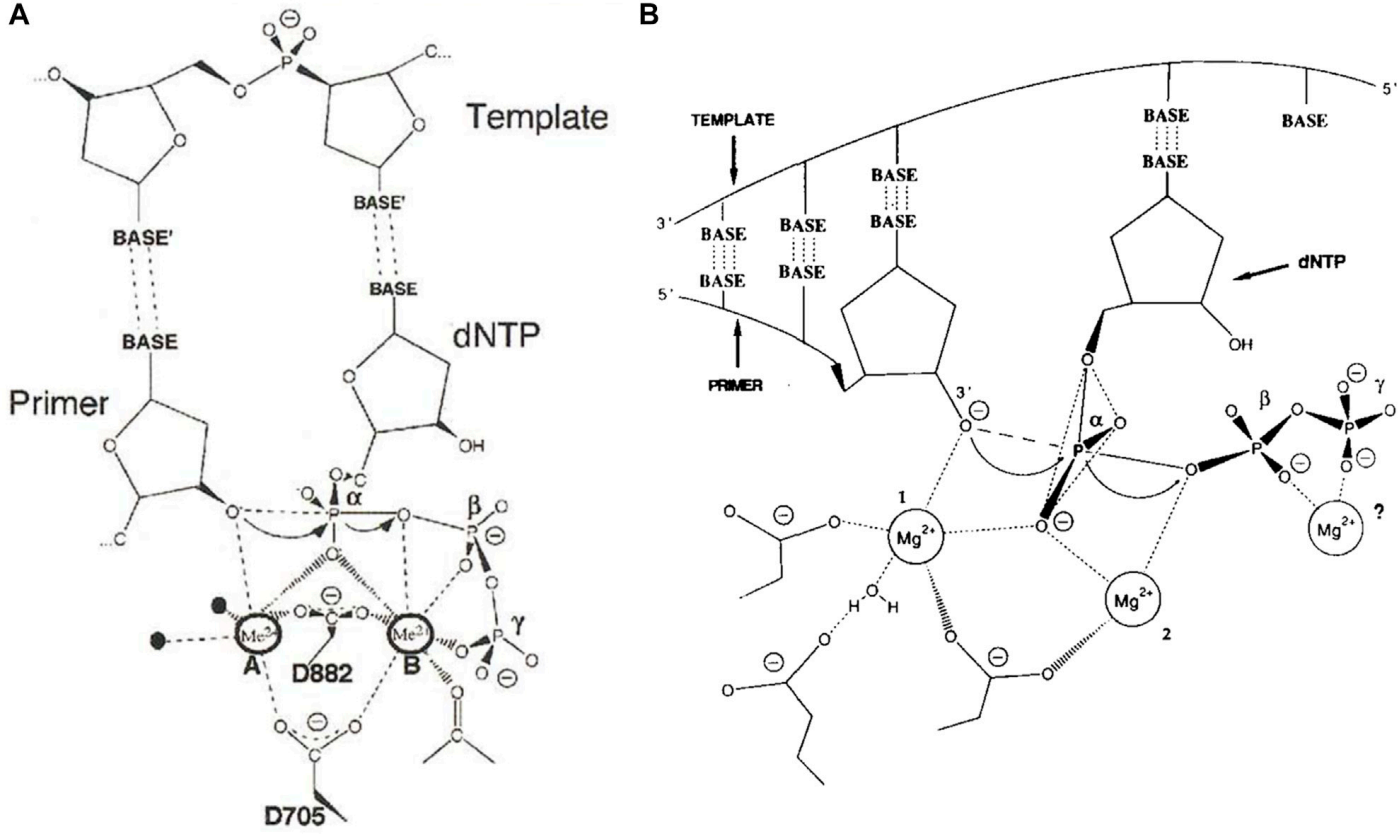
The two-metal-ion catalytic mechanism for nucleic acid polymerases **(A)** Two universally conserved aspartate residues (numbering is for the Klenow fragment of *E. coli* DNA polymerase I) coordinate two divalent cations, usually Mg^2+^, that facilitate deprotonation of the 3′-hydroxyl of the primer and stabilize the negative charges on the phosphates of the nucleotide and formation of the pentacovalent transition state. Figure is based on the structure of T7 DNA polymerase (Doublié et al., 1998) and is reproduced from Brautigam and Steitz (Brautigam and Steitz, 1998) with permission **(B)** The original mechanism proposed for polymerization based on the structure of the 3′-to-5′ exonuclease active site of Klenow fragment (Beese and Steitz, 1991). The possibility of a third metal ion binding to the beta and gamma phosphates was considered. Figure reproduced from Steitz (Steitz, 1993) with permission.

The two-metal-ion catalytic mechanism for polymerases was proposed in 1993 by Thomas A. Steitz (Steitz, 1993), based on structural studies of the 3′-5′ exonuclease active site of the Klenow fragment of *E. coli* DNA polymerase I (Beese and Steitz, 1991; Beese et al., 1993) and mutagenesis of the polymerase active site (Polesky et al., 1992). Structural support for this mechanism came over the next several years, when crystal structures were determined with primer-template DNA and dNTP poised for catalysis at the polymerase active sites of several different DNA polymerases and HIV-1 reverse transcriptase (Pelletier et al., 1994; Doublié et al., 1998; Huang et al., 1998; Li et al., 1998). These and subsequent structures show that polymerases have two absolutely conserved aspartate residues that coordinate two divalent cations in the polymerase active site (Figure 1A), demonstrating that the two-metal-ion catalytic mechanism is also applicable to DNA synthesis (Brautigam and Steitz, 1998).

More recently, however, a three-metal-ion polymerase mechanism has been proposed based on time-resolved crystallographic studies of translesion and repair DNA polymerases eta and beta, respectively (Nakamura et al., 2012; Gao and Yang, 2016). In fact, Thomas Steitz had initially considered that a third metal ion might be involved (Figure 1B). In the first article in this eBook, Wang and Konigsberg review the effects of pH and Mg^2+^ concentration on high-fidelity DNA polymerase activity and argue that the three-metal-ion mechanism may not be universal to all polymerases, suggesting instead that the third metal ion stabilizes pyrophosphate binding after catalysis and thus slows product release (Wang and Konigsberg, 2022).

The review of viral RNA-directed RNA polymerases by Gong highlights the complexities of the nucleotide incorporation cycle, including conformational changes that accompany nucleotide binding and pyrophosphate release, polymerase translocation along the template that is required for processive synthesis, and events outside of the standard catalytic cycle that can impact the fidelity of replication (Gong, 2021).

Carvajal-Maldonado provide a comprehensive review of the other catalytic activities that are frequently associated with DNA polymerases: 3′-5′ exonuclease proofreading that increases replication fidelity, structure-specific 5′-nuclease activity required for Okazaki fragment maturation during lagging strand synthesis, 5′dRP lyase activity required in the base excision repair pathway, and 3′-end-trimming and single-strand extension involved in double-strand break repair (Carvajal-Maldonado et al., 2021).

Kaszubowski and Trakselis focus on the challenges of coordinating multiple polymerases during translesion synthesis where a high-fidelity replicative DNA polymerase encounters DNA damage and is replaced by one of a number of possible specialized enzymes with lower fidelity that allow replication of damaged DNA (Kaszubowski and Trakselis, 2021). The review compares passive and active mechanisms for the handoff of DNA between the polymerases and discusses the role of the sliding clamp processivity factor.

Nucleic acid synthesis is a highly dynamic process and the review by Millar emphasizes how single-molecule Fluorescent Energy Transfer techniques have been able to elucidate the conformational changes that occur during the *E. coli* DNA polymerase I nucleotide incorporation cycle and as the DNA transitions between the polymerase and nuclease active sites–processes that are difficult to resolve using more static structural methods (Millar, 2022).

The original research articles in this eBook emphasize how much there still is to learn about the wide variety of polymerases. Frey et al. use X-ray crystallography and molecular dynamics simulation to describe new non-nucleoside inhibitors of HIV-1 reverse transcriptase that are effective to mutants that are resistant to previously designed compounds (Frey et al., 2022). This work highlights the importance of polymerases as drug targets but also emphasizes the importance of understanding the entire nucleotide incorporation cycle, both kinetically and structurally, in the drug development process.

The work by Park et al. demonstrates that mitochondrial DNA polymerase gamma is capable of efficiently bypassing a CPD lesion at physiological concentrations of Mn^2+^ (Park et al., 2022). This ability is specific to polymerase gamma, not a general property of A-family DNA polymerases, underscoring the diversity of polymerases and emphasizing the role of cellular conditions in regulating activity.

Vaisman et al. address the evolutionary diversity of the translesion DNA polymerases. Biochemical characterization of the four Y-family polymerases (eta, iota, kappa and Rev1) from a lower eukaryote shows that their major properties are very similar to those of their human homologs (Vaisman et al., 2021). This work indicates that polymerase iota evolved earlier than previous sequence analysis had suggested, raising the question of what critical role this enzyme plays in both lower and higher eukaryotes.

In the final research article in this volume, Park et al. present two structures of phage RB69 DNA polymerase in open binary and partially closed ternary complexes that are distinct from previous structures of this enzyme (Park et al., 2021). Since these structures exist in a single crystal form, they suggest that initial binding of the correct incoming nucleotide and the second divalent metal ion are much weaker than expected.

The various articles in this issue demonstrate that, despite decades of seminal work on polymerases in replication and transcription, there are still many unknowns that require future research. We hope this issue will inspire graduate students and postdocs to devote their research to studying these fascinating processes that are fundamental to all life.

We wish to dedicate this special polymerase issue to the memory of Tom Steitz who was a mentor to all of us, both directly (JDP and YWY) and indirectly (IL). His pioneering structural work, insights into catalytic mechanism, and deep appreciation of the connections between biological structure and function continue to inspire our own research. We miss him deeply.
